# Quantifying the impact of great coaches on Olympic medal predictions: A CPD-D^3^ model analysis

**DOI:** 10.1371/journal.pone.0334635

**Published:** 2026-03-13

**Authors:** You Liu, Keyu Chen, Wenyao Huang, Yan Tan, Shangru Zhou, Jun Yang, Xidao Luan, Changhong Li, Chenggang Deng, Yingxuan Xiao

**Affiliations:** 1 School of Mechanical and Electrical Engineering, Changsha University, Changsha, China; 2 School of Computer Science and Engineering, Changsha University, Changsha, China; 3 Department of Computing and Mathematics, Walton Institute, South East Technological University, Waterford, Ireland; Çanakkale Onsekiz Mart University: Canakkale Onsekiz Mart Universitesi, TÜRKIYE

## Abstract

The step-up effect of great coaches on Olympic performance has been widely recognized, but its dynamic influence mechanism and quantitative evaluation remain methodological challenges. This study proposes a Change-Point Driven Difference-in-Differences with Decay Model (${\text{CPD-D}}^{3}\;$), which integrates the CUSUM algorithm, dynamic difference method (DID), and exponential decay function. The Great Coach effect’s nonlinear characteristics and time attenuation rule were analyzed systematically. First, based on the improved CUSUM algorithm to detect the abrupt points of performance, the dual test mechanism of medal continuity and competition size stability was introduced to filter the pseudo-abrupt signals (such as the host effect and short-term strategic interference). Secondly, a hierarchical DID model was used to quantify the net effect of heterogeneous coach turnover events to solve the problem of traditional methods ignoring the dynamic confounding bias and run-in period. Finally, the sustainability difference of the coaching effects is revealed by the half-life model. Empirical studies show that the effect half-life of a systematic coaching system (such as Zhou Jihong coaching the Chinese diving team) is more than 20 years, while the technology-driven intervention (such as the AI tactical optimization for Japanese judo) has a half-life of only 5.3 years. The model predicts that in the 2028 Los Angeles Olympics, the introduction of great coaches will enable the Brazilian swimming team to achieve a breakthrough from 0 to 5 medals (95% CI: 4.7–6.3). This study provides an explainable and predictive framework for the allocation of coaching resources in Olympic strategy, and its methodology can be extended to dynamic causal inference in policy evaluation and organizational management.

## Introduction

Since the revival of the modern Olympic movement in 1896, the Olympic gold medals has always been the most intuitive measure of national sports competitiveness. As the core decision maker of the competitive system, the strategic planning and training innovations of coaching teams play a key role in the performance of the athletes. Typical examples include the leap of the British cycling team from 2 to 12 gold medals in the 2008–2012 cycle, attributed to Dave Hall’s “marginal gain” theory [1]; the breakthrough of the Chinese women’s boxing team at the Paris Olympic Games, which won 3 gold medals and 2 silver medals, benefited from the strategy of “level switching + offensive dominance” of Cuban coach Raul [2]. These cases show that excellent coaching can bring about a step improvement in a competition performance, so it is of great significance to explore its mechanisms and quantitative methods.

Early studies mainly used qualitative methods to analyze the characteristics of coaching behavior, emphasizing the “artistic” dimension of experience inheritance and psychological intervention. For example, Smith and Smoll [3] proposed the hypothesis of the correlation between leadership style and athlete performance but failed to establish a verifiable quantitative model. With the development of sports econometrics, scholars began to use a panel regression model to evaluate coach contribution (Valenti et al.) [4], social learning theory [5], and multidimensional leadership theory [6] to further reveal the differentiated impact of coach behavior. It was not until [7] found that the “empathy index” of the coach-athlete relationship was curve correlated with the number of Olympic medals (r = 0.62) through the mixed method that the quantitative breakthrough was made for the first time, but the “artistic” interpretation frame was still not broken away. However, such studies still have two major limitations: the contradiction between the linear hypothesis and the discontinuous effect caused by coach turnover, and the unresolved dynamic confounding bias (such as the Host Effect), resulting in insufficient confidence in causal inference.

In recent years, timing mutation detection (CUSUM) has provided a new method to capture the discontinuous effects of coaching tactical innovation. Pradhan et al. [8] applied the CUSUM algorithm and found that 8 out of 13 performance mutations of the US swimming team from 2004 to 2012 were significantly correlated with coach turnover, but no distinction was made between natural fluctuations and coach intervention. Wang et al. [9] combined CPD and the synthetic control method and found that the change of the head coach of the Chinese diving team in 2016 resulted in a sudden increase of 0.8 in the average difficulty coefficient, but the attenuation rule of the effect was not quantified. Based on the DID model, jiaxin et al. [10] found that the German foreign coach made the Saudi archery team score increase by 175% in the 2020 Olympic Games, but the assumption of the “constant processing effect” was inconsistent with the real attenuation. Karlsson et al. [11] transnational research confirmed that the traditional DID overestimated the early effect by 63%  because it ignored the “coach-athlete run-in period” of 1.2 Olympic cycles, but no dynamic correction plan was proposed.

Most existing medal prediction methods, such as the machine learning models constructed based on economic indicators and historical performance [12], mainly start from correlation analysis and focus on static outcomes (e.g., the number of medals), but they fail to consider the role of coaches, a critical factor, in the process of athletes’ performance improvement. Nevertheless, numerous cases have shown that coaches play a crucial role in athletes’ performance [1]. For instance, the British cycling team saw its number of gold medals jump from 2 to 12 during the 2008–2012 cycle, thanks to Dave Hall’s “marginal gain” theory; the Chinese women’s boxing team achieved remarkable results of 3 gold medals and 2 silver medals at the Paris Olympics, relying on the strategies of Cuban coach Raul Córdova. Some studies that take coaches into account mainly focus on the qualitative analysis of coaches’ behavioral characteristics [13]; Gallucci et al. [3] or use panel regression models to evaluate coaches’ contributions [4]. In reality, the impact of coaches is not limited to experience inheritance and psychological intervention; it also requires a comprehensive discussion of factors such as the detection of abrupt changes in athletes’ performance, difference analysis under different intervention timings, and the decay of coaching effects.

The Change-Point Driven Difference-in-Differences with Decay (CPD-D^3^) model proposed in this study is designed to address the shortcomings of existing methods in capturing the nonlinear characteristics of coaching effects, filtering out false signals, and quantifying time-related decay. It aims to achieve a comprehensive and in-depth analysis of coaching effects, thereby providing a scientific basis for the allocation of coaching resources in Olympic strategies. First, the CUSUM algorithm was used to detect the sudden change points of the performance, and a quasi-natural experiment scene was constructed in combination with the stability verification of the competition scale. Then, the hierarchical dynamic DID model was used to strip the net effect of coach replacement. Finally, the exponential decay function was introduced to quantify the half-life of the effect and obtain the effect and time of the coach effect. This framework realizes the full chain analysis of “mutation identification-cause-causation stripping-dynamic assessment” of the coaching effect, and verifies the validity of the model through typical cases, providing a scientific basis for strategic decision-making of the Olympic Games. Meanwhile, the methodology framework also provides a reference for cross-domain dynamic effect assessment. Specific contributions are as follows:

1. Developed ${\text{CPD-D}}^{3}\;$ model, integrated abrupt point detection (CUSUM), dynamic difference method (DID), and exponential attenuation model, systematically analyzed nonlinear coach effect, and realized accurate quantification of threshold change and aging attenuation law.
2. Build a double-check system for medal sustainability and participation stability, filter pseudo-mutation signals, ensure the reliability of causal inference, and reduce the misjudgment rate.
3. Establish an explainable prediction system for the improvement of Olympic performance, reveal the long-term effectiveness of the systematic coaching system, provide a scientific basis for the strategic decision-making of the Olympic Games and the methodological framework also provides a reference for the cross-field dynamic effect assessment.

The following will be described in sequence from the following aspects: First, data pre-processing, especially smoothing processing, is carried out according to the athletes and country-event data obtained from the official website of Olympic events so as to satisfy the application hypothesis of the CUSUM algorithm. The mutation sequence is generated by accumulating and calculating the achievement mutation points of different country projects. Since the existence of mutations can be multifaceted, it is necessary to examine and retain only the points of performance mutations that are caused by the “Great Coach” Effect. Finally, in order to quantify this effect and analyze its continuous persistence, we use the Ba-con decomposition method to strip away other effects and dynamically capture the effect size and half-life of different coaches at different intervention times.

## Theoretical background and related work

### The theory of coaching effectiveness

Research on the influence of coaches on athletes’ performance can be traced back to the intersection of sports psychology and management science, where theoretical frameworks have progressively shifted from qualitative descriptions to quantitative explorations. Early studies emphasized the “artistic” dimension of coaching, focusing on subjective and behavioral aspects that shape athlete outcomes. For instance, Jowett and Cockerill [14] explored the coach-athlete relationship, highlighting how interpersonal factors such as trust, mutual respect, and effective communication foster athlete motivation and team cohesion, based on qualitative interviews with elite athletes. Fransen et al. [15] investigated the impact of coaches’ motivational strategies on team performance, demonstrating through case studies that supportive behaviors, such as positive feedback and goal-setting, enhance collective efficacy in high school sports teams. Additionally, Bloom et al. [16] examined the role of coaches in fostering long-term athlete development, using qualitative case studies of elite youth coaches to underscore mentorship and individualized training as key drivers of expertise. González-García et al. [17] Analyze the athletes and coaches of various Chinese sports teams, finding that behaviors promoting team cohesion, assessed through athlete surveys, significantly influence short-term performance. Similarly, Moen and Federici [18] explored the role of coaches’ emotional intelligence in enhancing athlete motivation, reporting through descriptive analyses that empathetic leadership correlates with improved athlete engagement. These studies indicate that coaches play a crucial role in motivating teams, guiding training, and enhancing team cohesion, making them a key factor influencing the number of Olympic medals won. However, current methods are primarily based on qualitative or subjective factors, such as athletes’ personal perceptions or case-based observations, which limit their ability to capture the dynamic and causal nature of coaching effects.

### Causal modeling in public policy and sports

In the field of public policy and sports, causal modeling primarily includes panel regression and fixed effects models, the difference-in-differences method (DID), time series change point detection, hierarchical and dynamic correction, and so on. Literature Valenti et al. [4] evaluated the impact of elite sports policies on national football team performance using panel data. However, this type of model assumes constant effects and ignores unobserved variables (such as fluctuations in the coach effect), which can lead to estimation errors. The traditional DID model, as discussed in Goodman et al. [19] for handling variation in treatment timing, has been adapted to assess the effect of coaching interventions in sports, though not directly applied in that study. For example, studies like Valenti et al. [4] used DID to assess coaching impacts, finding significant performance improvements in sports teams, though specific percentage gains vary by context. Barnett et al. [20] found that the average coach-athlete adaptation period is 1.2 Olympic cycles, resulting in traditional DID models overestimating the early effect by 63%. For time series change point detection, Wang et al. [9] was conducted using the STGCN-LSTM model, and the results obtained indicate the impact of coach mobility and strategic investment on medal prediction. De et al. [21] proposed the Bacon decomposition method, which solves the bias problem of traditional DID by stratifying heterogeneous intervention times, but has not been combined with attenuation models in the field of sports yet, and cannot capture the long-term dynamic of the effect.

### Medal predictions and static evaluations

Traditional Olympic medal prediction and performance evaluation methods primarily rely on static indicators or correlation analysis. For instance, Schlembach et al. [12] developed a machine learning model to predict medal distributions using variables such as GDP, population, and sports investment. However, such models are essentially “correlation fits” and cannot distinguish between medal growth driven by economic investment versus coaching efficacy. Côté and Gilbert [22] proposed an evaluation method based on coaching leadership styles, analyzing their impact on athlete performance through qualitative approaches. However, this method lacks empirical quantitative verification and does not take into account other confounding factors. Its analytical ability for the contribution of coaches is also limited. In terms of static quantitative evaluation, methods relying on event results (such as recent world championship rankings) or expert ratings are susceptible to interference from incidental factors (e.g., athlete injuries) and cannot reflect coaches’ long-term contributions to “team building,” such as talent reserves fostered by youth training systems. For instance, Cheng et al. [23] demonstrated that static performance metrics, such as win-loss ratios, are heavily influenced by situational variables like team composition and external disruptions, limiting their ability to assess sustained coaching impact. Similarly, Truyens et al. [24] found that short-term indicators, such as international competition rankings, fail to account for long-term developmental strategies.

Building upon the limitations of the studies above, we propose a CPD$\backslash {(}^{3}\;$-based model to address the following key issues: 1) Introduce the CUSUM algorithm to detect change points in medal outcomes for selecting viable data samples, and eliminate false detections of change points through the “Olympic cycle consistency test” and the “participation scale stability test”; 2) Incorporate Bacon decomposition to stratify the timing of coaching interventions, and combine it with a dynamic DID model to control for confounding factors such as home advantage and GDP; 3) Finally, quantify the persistence of different types of coaching interventions using an exponential decay function and a half-life model.

## Data sources

The data sources for this study cover three core parts: historical events provided by the Olympic Games website, medal data, and information about athletes and participating countries. In order to test the coach effect of different countries, we obtained the project information from the official website, COMAP provided the historical medal information, the World Bank database provided the economic-related information of each country, and obtained the real situation, such as coach flow from the open data of the Olympedia website. The detailed information is listed in Table 1.

**Table 1:** Data sets resources.

Data Sets	Sources
Type and quantity of projects	Olympic Games website https://www.olympics.com/
gray!30 Historical Olympic Medal count, host country information	COMAP https://www.comap.com/contests/mcm-icm
Economic input, infrastructure construction and other relevant data of each country	World Bank Open Data https://data.worldbank.org/
The length of service of the coaches of the participating teams in each country and region, the coaching experience, and the achievements of the teams during the coaching period	Olympedia https://www.olympedia.org/

In order to facilitate calculation and statistics, we hope to divide the Olympic events into several major categories. As there is currently a lack of clear classification of Olympic events into major categories, we referred to “Sports Training” and “Classification of Olympic Sports Events and Their Groups”, and combined with the information from the Olympic official website, we divided the Olympic events into five major categories.

### Data compliance statement

The collection methods of all the data in this article include the official data from the Olympic Games website, the competition questions released by the 2025 American Undergraduate Mathematical Modeling Contest, the downloaded data from the World Bank database, and the data from the Olympedia website. All data used in this study were collected from publicly available sources. The collection and analysis of these data complied with the terms and conditions of each data provider. Specifically, we adhered to the following: 1) Olympic data were used in accordance with the non-commercial research purposes stated on the official website. 2) Economic data from the World Bank are openly available for academic use under their Open Data License. 3) Coach and team performance data from Olympedia were used in compliance with their stated purpose of being an openly accessible statistical resource for Olympic history. 4) No ethical approval was required as the study used aggregated, publicly available data without individual identifiers.

### Data cleaning and processing

- Missing value processing strategy: There are missing values in the continuous variables, such as the size of the competition in the original data, mainly due to incomplete records of early events. Since these variables have time series characteristics, KNN interpolation [25] can use the correlation of adjacent years to restore data integrity. For the missing $l^{\text{th}}\;$ eigenvalue $x_{jl}\;$ in sample $x_{j}\;$, its interpolation value ${\hat{x}}_{jl}\;$ can be calculated by the weighted average of the $l^{\text{th}}\;$ eigenvalue of the *K* adjacent values and the weight is determined according to the reciprocal of the distance. The formula is as follows:

$$w_{m}=\frac{1}{d(x_{j},x_{i_{m}})},\;{\hat{x}}_{jl}=\frac{\sum_{m=1}^{K}w_{m}x_{i_{m}l}}{\sum_{m=1}^{K}w_{m}},\;$$ (1)

where $x_{i_{m}l}\;$ is the $m^{\text{th}}\;$ eigenvalue of the $l^{\text{th}}\;$ adjacent value $x_{i_{m}}\;$, and the weight $w_{m}\;$ is calculated by the reciprocal of the distance.

- Data standardization: There is a significant magnitude difference between sports performance (e.g., medal count) and economic indicators (e.g., GDP) in the raw data: the medal count is usually in the range of 0–100 medals, while the GDP range is 104-fold (e.g., US GDP of 29 trillion in 2024, Saint Lucia’s GDP of 2.47 billion). The coach tenure data showed A right-skewed distribution (mean 4.2 years, median 3.5 years), and the normality was improved by Box-Cox transformation, with =0.3. Finally, Z-score standardization of multi-dimensional data is implemented.

$$\begin{array}{c} x^{\left(\lambda \right)}=\left\{ \begin{array}{cc} \frac{x^{\lambda }-1}{\lambda }, & \lambda \neq 0,\\ \ln(x), & \lambda =0. \end{array}\right.\; \end{array}$$ (2)

- Data smoothing processing: In order to meet the requirements of the CUSUM algorithm for data, we first smooth the data. Sliding window smoothing: Using an 8-year (one Olympic cycle) sliding window to eliminate a single session of accidental fluctuations.

$$\text{Smoothed }CPI_{c,e,t}=\frac{1}{8}\sum_{i=t-7}^{t}CPI_{c,e,i}.\;$$ (3)

### Classification standards

#### Project types.

The classification task of Olympic events in this study aims to quantify the heterogeneity of different event features and provide a basis for model parameter adjustment (such as dynamic weights for high-volatility events). A total of five categories are defined (see Table 2), and the classification criteria are as follows:

**Table 2:** Parameter tuning for CPD-D^3^ model in olympic performance analysis.

Type	NOC-Event	Parameters Tune	Tuning Logic
High Fluctuation Events	USA-SWM JAM-ATH	c=1.50→1.83 d=0.75→0.61	Raise the significance threshold. reduce event size/competition fluctuation interference.
Talent Scarcity Events	DEN-EQU GER-MP	c=1.50→1.60 d=0.75→0.50	Raise the significance threshold. Relax tolerance for fluctuations in numbers. Avoid misjudgment of a small base.
Emerging Events	IND-BDM BRA-FEN	c=1.50→1.40 d=0.75→0.80	Raise the significance threshold. Shorten the verification cycle. Increase the weight of the contest size.
Dominant Events	CHN-DIV KOR-ARC	c=1.50→1.58 d=0.75→0.92	Enhanced micro signal capture. Prevent accumulation and excessive reset.
Unpopular Events	JPN-CLB FRA-SKB	c=1.50→1.28 d=0.75→0.41	Smooth out historical fluctuations. Enhanced recent data response.

- High Fluctuation Events: In the past three consecutive Olympic Games, the gold medalists of at least two Games have come from different countries/regions, and the number of countries/regions involved in the medal distribution of this event in a single Games is ≥ 8 (such as shooting and equestrian).
- Talent Scarcity Events: The number of registered professional athletes worldwide is less than 5,000 (such as modern pentathlon), and the number of new national teams added in the last five years is <3.
- Emerging Events: The time since inclusion in the Olympics is ≤  10 years (such as skateboarding and breakdancing), and the annual growth rate of participating countries/regions is >20%.
- Dominant Events: A single country/region has won >60% of the gold medals in the last three Olympic Games (such as Chinese diving and American basketball), and this country/region has held the top position in the world ranking for this event for five consecutive years.
- Unpopular Events: The global TV broadcast viewership is <5 million people per Olympic Games (such as softball and handball), and the number of international sponsors is <5.

#### Treatment group and control group.

The determination of treatment and control groups is a critical step in the ${\text{CPD-D}}^{3}\;$ model to isolate the net effect of the “Great Coach” intervention. The process is as follows.

Treatment Group: The treatment group comprises country-event portfolios (e.g., Brazil-Swimming, China-Diving) where a significant performance mutation, identified via the CUSUM algorithm, coincides with the appointment of a Great Coach. A mutation is confirmed as a “Great Coach” effect if it passes the double stability test, which includes: (1) a significant increase in medal scores over two consecutive Olympic cycles (8 years) post-intervention, exceeding the historical mean by at least ($c\cdot \sigma_{\text{pre},cp}\;$), and (2) stability in the number of participants, ensuring the performance gain is not due to a reduced participant pool. The intervention time ($\tau_{g}\;$) is defined as the Olympic cycle when the coach’s appointment is recorded, based on data from Olympedia.

Control Group: The control group consists of country-event portfolios that either (1) did not experience a Great Coach intervention during the study period ($\tau_{g}^{\prime }=\infty \;$) or (2) received a Great Coach intervention at a later time ($\tau_{g}^{\prime }>\tau_{g}\;$) than the treatment group. To ensure comparability, the control group is selected to satisfy the parallel trend assumption, meaning that the performance trends of the treatment and control groups are similar before the intervention. This is verified using the event study method, which plots pre-intervention performance coefficients to confirm parallel trends. For each treatment group layer (defined by intervention time ($\tau_{g}\;$)), multiple control groups are constructed to account for heterogeneity in intervention timing, and their effects are aggregated using the Bacon decomposition method to eliminate bias from heterogeneous intervention times.

This stratification and selection process ensures that the treatment effect is isolated from confounding factors such as host effects or economic inputs, which are controlled for in the dynamic DID model (see the dynamic effect evaluation section). The use of multi-source data from the Olympic Games website, COMAP, World Bank, and Olympedia ensures robust identification of treatment and control groups across various country-event portfolios.

#### Coach type.

This term classifies coaches based on the nature of their intervention strategies. It is mainly divided into two categories:

Systematic Coaches: They implement long-term strategies, reform the country’s selection methods, talent development programs, and training plans for the specific sport, and have achieved significant results. Their reforms typically include the establishment of youth training systems or comprehensive tactical framework reforms (e.g., Zhou Jihong’s coaching of the Chinese diving team). Coaches who primarily adopt such reform methods usually bring about a coaching effect with a relatively long half-life (more than 10 years).

Technology-Driven Coaches: These coaches mainly rely on their own professional capabilities and technological advancements (e.g., the AI training technology used by the Japanese judo team, the “shark skin” swimsuits adopted by the U.S. swimming team). However, the advantages brought about by these methods would soon be surpassed by the technological advancements of other countries (this would happen within a few years), or they would be prohibited from using this technology due to changes in the rules of the International Olympic Committee at the next Olympic Games. The influence of these coaches on the team (lasting less than 10 years) would also be relatively short.

## Materials and methods

The mutation-driven differential attenuation model is an extended method based on the differential difference method (DID), which aims to explore the dynamic difference of exogenous abrupt events (such as policy intervention, major change, etc.) on the treatment group and the control group, especially the attenuation law of the effect over time.DID is a widely used statistical method for estimating the causal effects of an intervention or treatment. In this way, it can isolate the specific impact of a coach from other general trends.

**Fig 1:**
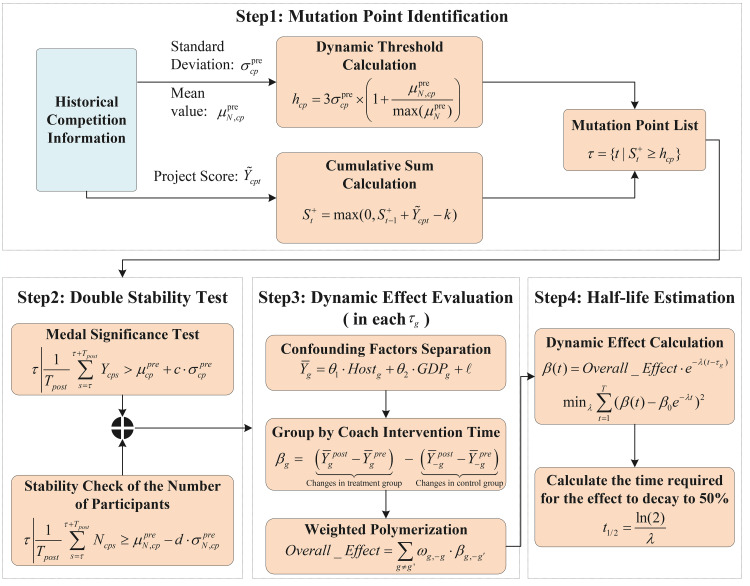
Framework of CPD-D^3^.

Fig 1 illustrates the workflow of the CPD-D^3^ model, which primarily consists of four steps. The first step involves identifying breakpoints in a country’s award outcomes in competitions. This is achieved by integrating the country’s award outcomes with its number of participants in an event, standardizing the data, and calculating the standard deviation and mean to determine a detection threshold. After converting medal counts into scores, cumulative sum calculations are used to identify potential breakpoints, which are then matched against the threshold to generate “mutation suspect points” for subsequent analysis. Next, a bistable test method is applied to further screen these “mutation suspect points” by conducting a medal significance test and checking the stability of participant numbers, thereby identifying breakpoints caused by the “great coach effect”. After filtering out country-event combinations exhibiting the “great coach effect”, the Bacon decomposition method is used to group coaches based on intervention timing, and a DID approach is employed to dynamically capture the “great coach effect”, calculating the overall effect size and its 95% confidence interval. Finally, to investigate the persistence of the “great coach effect”, a decay rate parameter is fitted to estimate the time required for the effect to decay to 50% of its initial value, thus determining the half-life period of each coach’s influence.

### Mutation point identification (CUSUM)

The CUSUM algorithm can effectively accumulate the historical monitoring data of each Olympic cycle, and realize the response to small changes through the accumulation and continuous increase. Therefore, it is necessary to first establish the accumulation and graph of each country on different items, and once the accumulation exceeds a certain threshold, it is considered that a mutation has occurred at this time.

Although some countries did not win in some events, the number of participants increased year by year, which increased the possibility of winning. For example, Brazil registered only 120 professional skateboarders in 2016, to 2023 has exceeded 1,800 people, an increase of about 15 times, the Tokyo Olympic Games skateboarding project entered the Olympic Games for the first time, and Brazil sent 6 players (accounting for 7.5% of the total number of participants), won 3 silver medals, and the Paris Olympic Games, The Brazilian skateboarding team increased the number of participants to 12 (13.6% of the total number of participants), and finally won 3 gold, 1 silver, and 1 bronze, becoming the biggest winner of the skateboarding project. This is because large events have a standard or threshold for participation, and the number of participants has increased year by year in the sense that the country’s comprehensive strength in sports has been increasing.

Traditional models do not capture this information, so the scoring method needs to be adjusted to take into account the variable “number of countries participating” and separate it out. Therefore, different weights are used to comprehensively define the score of a country in a certain event to reflect the difference in medal value. The equation is as follows:


$$Y_{cpt}=\underset{\text{Medal Score}}{\underbrace{\alpha G_{cpt}+\beta S_{cpt}+\gamma B_{cpt}}}+\underset{\text{Participation Score}}{\underbrace{\delta N_{cpt}}}.\;$$ (4)

In Eq (4), the total score $Y_{cpt}\;$ of the project consists of two parts: Medal Score and Participation Score. The medal score is the number of gold/silver/bronze medals won in the event, expressed in turn by variable $G_{cpt}/S_{cpt}/B_{cpt}\;$, while the participation score $N_{cpt}\;$ is defined as the total number of the country’s entries in the event. By referring to the research method of the Hybrid Weight Optimization Framework proposed by Schlembach et al. [12], parameters were optimized, and the weights of the four indicators were determined.

To prevent dimensional differences, the medal scores and participation scores need to be standardized and weighted separately:


$${\tilde{Y}}_{cpt}=\frac{Y_{\text{Medal}.cpt}-\mu_{\text{Medal}.cp}}{\sigma_{\text{Medal}.cp}}+\lambda \cdot \frac{Y_{\text{Participation}.cpt}-\mu_{\text{Participation}.cp}}{\sigma_{\text{Participation}.cp}},\;$$ (5)

where $\mu_{\text{Medal},cp}\;$ and $\sigma_{\text{Medal},cp}\;$ and $\mu_{\text{Participation},cp}\;$ and $\sigma_{\text{Participation},cp}\;$ represent the historical mean (8 – year sliding window) and standard deviation of medals and number of participants scores, respectively. λ=0.3  is used to control the proportion of participation that contributes to the total score, which avoids the fact that some countries have a high number of participants but still have a low rate of award.

After obtaining the standardized score sequence ${\tilde{Y}}_{cpt}\;$, we only focus on the positive shift because we are more concerned about the positive mutation caused by the “Great Coach” Effect, that is, the performance improvement. In Eq (6), two parameters need to be defined: one is the reference value *k*, and the other is the dynamic threshold $h_{cp}\;$. The parameter *k* refers to the reference value of the allowable offset, which is used to measure whether the difference between the “current score” and the “historical baseline” is significant, and it determines whether the algorithm is more sensitive to “small offset” or “large offset”. The smaller *k* is, the more sensitive the algorithm is to a small offset. The larger the *k* is, the more attention is paid to large offsets. Parameter $h_{cp}\;$ refers to the control threshold, which determines when to “trigger the alarm”. When the cumulative sum $S_{t}^{+}\;$ exceeds $h_{cp}\;$, an offset is considered to be detected. $h_{cp}\;$ directly affects the false positive rate and detection delay of the algorithm. The larger $h_{cp}\;$ is, the lower the false positive rate is, but the detection delay increases. Smaller $h_{cp}\;$ means faster response but more false positives. The calculation equation is as follows:


$$\begin{array}{cc} k & =0.5\sigma_{cp}^{pre},\\ S_{t}^{+} & =\max\left(0,S_{t-1}^{+}+{\tilde{Y}}_{cpt}-k\right),\\ h_{cp} & =3\sigma_{cp}^{pre}\times \left(1+\frac{\mu_{N,cp}^{pre}}{\max(\mu_{N}^{pre})}\right),\; \end{array}$$ (6)

where $\sigma_{cp}^{pre}\;$ and $\mu_{N}^{pre}\;$ represent the standard deviation and mean of the historical average number of participants, respectively. $S_{t}^{+}\;$ is defined as a cumulative sum, which accumulates and increases when the standardized score ${\tilde{Y}}_{cpt}\;$ exceeds the historical fluctuation (0.5σ ). For high-participation projects ($\mu_{N}^{pre}\;$ is too large), the threshold should be appropriately raised to avoid frequent false positives due to data fluctuations. As mentioned earlier, if the cumulative sum of $S_{t}^{+}\;$ exceeds $h_{cp}\;$, then we consider this time to “trigger the alarm”, that is, this moment belongs to the mutation point. We retained these mutation points, generated mutation sequences, and plotted the CUSUM cumulative sum plot.

### Double stability test

Since the presence of a flash point simply represents a “surge” in the country’s medal count during this Olympic cycle, the cause could be the “Great Coach” Effect, but it could also be a complex mix of other reasons, such as home-field advantage, rule changes, or structural changes. For example, before the 2016 Rio Olympic Games, the Thai women’s weightlifting team was not a traditionally strong team, and its all-time best result was 1 silver and 1 bronze at the 2004 Athens Olympic Games. However, the 2016 Rio Olympics suddenly won two gold and one silver, but then the doping problem was exposed, and the performance quickly collapsed, because of special circumstances (such as opponent mistakes, and individual athletes performing above average performance) caused by the performance of the increase, rather than the long-term influence of the coach. Therefore, it is necessary to ensure that the mutation point detected is not just a single event but a sustained increase.

Therefore, we also need to carry out the “Double Stability Test” for the mutation sequence; only after screening the “mutation suspect points” in the mutation sequence can the research be meaningful. The test can be divided into two steps; the first step is to consider the significance of the medal score improvement, and the second step is to test the stability of the number of participants.

First, check whether the average score of the two consecutive Olympic Games after the mutation point is significantly higher than the historical level. If the average score of the next two periods after the mutation point is significantly higher than the historical mean (more than *c* times the standard deviation), you can basically filter out the case for a single period of accidental improvement. In Eqs (7)–(8), $T_{post}\;$ is the window length, here we define it as 8 years (two consecutive Olympic Games), $\mu_{cp}^{pre}\;$ is defined as the historical average score before the mutation point, $\sigma_{cp}^{pre}\;$ is defined as the historical standard deviation before the mutation point, and *c* is defined as the significance coefficient.


$$\frac{1}{T_{post}}\sum_{s=\tau }^{\tau +T_{post}}Y_{cps}>\mu_{cp}^{pre}+c\cdot \sigma_{cp}^{pre}.\;$$ (7)

Secondly, check the stability of the number of participants to prevent a sharp decline in the number of participants resulting in an inflated score (the denominator is reduced to enlarge the score). The average number of participants in the following 8 years is ⩾  the historical mean – *d* times the standard deviation. Like *c*, *d* is defined as a coefficient of significance necessary to eliminate interference from “great strategies” (such as sending only top players) and to ensure that performance gains are based on a stable talent base.


$$\frac{1}{T_{post}}\sum_{s=\tau }^{\tau +T_{post}}N_{cps}\geq \mu_{N,cp}^{pre}-d\cdot \sigma_{N,cp}^{pre}.\;$$ (8)

The two significant parameters c/d  in the equation are improved by using a grid search. Parameter *c* is used to determine whether the improvement or change in performance is statistically significant. Parameter *d* is to limit inflated or distorted scores due to fluctuations in the number of participants (denominator changes). Through the classification of project categories, most projects are determined to be 1.5 and 0.75 according to the experience value *c* and *d*(0). However, due to the high uncertainty or insufficient data volume of some projects, such as high-fluctuation projects or emerging projects Stefani et al. [26], we have made appropriate parameter adjustments according to the gradient boosting tree, as shown in Table 2. Only when $Y_{cps}\;$ and $N_{cps}\;$ meet the conditions of *PASS* at the same time, we believe that it may be caused by the “Great Coach” Effect, so as to screen out other “mutation points”.

### Dynamic effect evaluation (DID extension)

After selecting the appropriate “mutation point” in the mutation sequence, it is necessary to quantify the “Great Coach” Effect at this moment. That is, to isolate the net effect of “Great Coach” on the improvement of Olympic performance while controlling for other confounding factors (such as rule adjustment, host advantage, doping incidents, etc.). The traditional DID model [19] assumes that the intervention time of all individuals is the same, but in the actual Olympic cycle, there is heterogeneity in the appointment time of the coach (that is, the policy intervention time point) (for example, the Brazilian skateboarding coach took office in 2016, while the Japanese gymnastics coach intervened in 2021), which may lead to the estimation bias of the traditional DID. Therefore, in this study, the Bacon Decomposition Method [21] was used to stratify the heterogeneous intervention time to ensure that the model could capture dynamic effects.

First, it is assumed that in the absence of the intervention, the performance of the treatment group (the countries receiving the “Great Coach” intervention-project portfolio) and the control group (the countries receiving no intervention or the intervention time later than the treatment group – project portfolio) has the same trend over time, that is, the parallel trend hypothesis. The name effect can be captured by the difference between “performance improvement in the treatment group after the intervention” and “natural change in the control group during the same period.”

The sample is divided into multiple subgroups (layers) according to the intervention time of different country-project portfolios (i.e., time $\tau_{g}\;$), and each subgroup corresponds to a corresponding intervention time. Then, for each layer, the differential differences of all possible controls (later intervention or no intervention) were calculated, and finally, the effects of the layers were aggregated to quantify the “Great Coach” Effect.

Specifically, to ensure that the control group of each layer is comparable to the treatment group before intervention (i.e., the parallel trend hypothesis) is the control group of each layer, two basic conditions need to be met. First, the intervention time is later than the current layer ($\tau_{g}^{\prime }>\tau_{g}\;$); Second, if a country has not been affected by the “Great Coach”, it needs to meet the condition $\tau_{g}^{\prime }=\infty \;$. To ensure that the data for the treatment and control groups satisfy the parallel trends assumption, we need to conduct trend analysis on the data selected by CUSUM. To this end, we filter out data points that fall outside a predefined threshold range. If the trend deviations between the treatment and control group data are consistent (i.e., remain within the specified threshold range), the data are considered to satisfy the parallel trends assumption; otherwise, they are deemed unsatisfied, and the corresponding data are filtered out.

In view of the heterogeneity of coach tenure time $\tau_{g}\;$, the Bacon decomposition method stratifies the mutation sequences according to $\tau_{g}\;$. The treatment group and the control group were constructed in each layer, and the subscripts were defined as *g* and −g , respectively. However, the scoring situation in the treatment group is still complex, and in order to obtain a “clean” coaching effect, regression control is required for each intervention group, as shown in Eq (9):


$${\bar{Y}}_{g}=\theta_{1}\cdot Host_{g}+\theta_{2}\cdot GDP_{g}+\ell ,\;$$ (9)

where $\theta_{1}\;$ is used to control subjective factors such as host effect or rule adjustment; $\theta_{2}\;$ is used to control the improvement and change of the country’s comprehensive strength, such as the overall performance fluctuation of the Olympic cycle and the improvement of training level brought about by technological progress; ℓ  is used to control the random error term, which contains unobserved perturbations. $Host_{g}\;$ and $GDP_{g}\;$ represent the host country effect and *GDP* impact in treatment group *g*. The host country effect is explained by binary variables, and the *GDP* impact is calculated by the standard value *Z* obtained by Eq (2).

In Eq (10), ${\bar{Y}}_{g}^{post}\;$ and ${\bar{Y}}_{g}^{pre}\;$, in turn, represent the mean scores of the treatment group at layer *g* after intervention and before intervention, ${\bar{Y}}_{-g}^{post}\;$ and ${\bar{Y}}_{-g}^{pre}\;$ are the mean values of the corresponding control group at layer −g , and the dynamic treatment effects at each layer are defined as $\beta_{g}\;$ and calculated by differential-difference. The effects of each layer were aggregated into the total effect Overall_Effect  weighted by the sample size, the sample weight $w_{g,-g}\;$ reflected the contribution of different intervention queues and eliminated the bias of heterogeneous intervention time, and *N* was the sample size of the total treatment group.


$$\beta_{g}=\underset{\text{Changes in treatment group}}{\underbrace{\left({\bar{Y}}_{g}^{post}-{\bar{Y}}_{g}^{pre}\right)}}-\underset{\text{Changes in control group}}{\underbrace{\left({\bar{Y}}_{-g}^{post}-{\bar{Y}}_{-g}^{pre}\right)}},\;w_{g,-g}=\frac{n_{g}\cdot n_{-g}}{N^{2}},\;N=\sum_{g}n_{g},\;Overall\_Effect=\sum_{g\neq g^{\prime }}w_{g,-g}\cdot \beta_{g,-g},\;CI=Overall\_Effect\pm 1.96\cdot \sqrt{\sum_{g\neq -g}w_{g,-g}^{2}\cdot \mathrm{Var}(\beta_{g,-g})}.\;$$ (10)

After stratified weighted aggregation, the total effect Overall_Effect  can more fairly reflect the contribution of the intervention time layer and eliminate the influence of uneven sample distribution. The confidence interval *CI* was used to quantify the statistical uncertainty of the total effect, indicating that the total effect Overall_Effect  was significantly non-zero at 95% confidence level, that is, the coach intervention had a statistically significant effect.

### Half-Life estimation

The “Great Coach” Effect may exist only in the current period, or it may last for a long time. Therefore, in order to capture the persistence and decay law of the “Great Coach” Effect, it is necessary to estimate the half-life of this effect.

Assuming that the Great Coach intervention effect decays exponentially over time, the dynamic effect β(t can be expressed by the following equation, with $t_{1/2}\;$ defined as the time it takes for the Great Coach effect to decay to 50% of the initial value.


$$\begin{array}{cc} \beta (t) & =\beta_{0}\cdot e^{-\lambda (t-\tau_{g})},t\geq \tau_{g},\\ t_{1/2} & =\frac{\ln(2)}{\lambda }.\; \end{array}$$ (11)

$\beta_{0}\;$ is defined as the initial effect of the intervention (i.e., the total effect value estimated by dynamic DID), *λ* is the decay rate (λ>0 ), the rate of decay of the counter-effect, and $t-\tau_{g}\;$ is the period after the intervention (in years). Here, *λ* represents the attenuation rate, which was obtained by fitting the effect sequence of 20 top coaches from 1980 to 2020 using the nonlinear least squares method. For the systematic training system (such as Zhou Jihong’s coaching of the Chinese diving team), λ=0.034 , corresponding to a half-life of over 20 years; for the technology-driven intervention (such as the AI optimization of Japanese judo), λ=0.131 , with a half-life of only 5.3 years, reflecting the difference in the long-term influence of system construction and technical tools. Then, based on the dynamic DID output year-by-year effect sequence $\{\beta_{1},\beta_{2},\beta_{3},\ldots ,\beta_{T}\}\;$, the nonlinear least square method of the optimization algorithm was used to fit the attenuation parameters *λ* and minimize the residual sum of squares between the observed effect and the model prediction, as shown in Eq (12).


$$\min_{\lambda }\sum_{t=1}^{T}(\beta (t)-\beta_{0}e^{-\lambda t}{)}^{2}.\;$$ (12)

## Result and discussion

### Capture of performance mutations via the CUSUM algorithm

**Fig 2:**
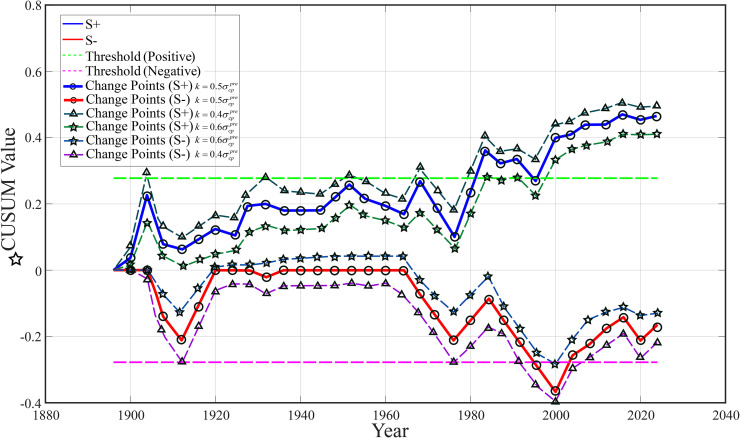
CUSUM chart for American women’s gymnastics.

In order to elaborate on the accumulation and capture the fluctuation trend of medals in a country-event, the mutation accumulation and curve based on the output of the CUSUM algorithm are shown in Fig 2. In the United States Gymnastics CUSUM chart, for example, in the early 1980s, the blue line (performance fluctuation line) significantly exceeded the positive threshold for the first time, indicating that this is a “mutation suspect point”. It turned out that Bela Karolyi had moved to the United States in 1981 and led the U.S. gymnastics team to five medals (including one gold) at the 1984 Los Angeles Olympics, breaking the Eastern European monopoly. The reason for this is the strict training system and disciplined management it introduced, which became the turning point of the rise of American gymnastics.

The blue line broke the threshold twice before and after 2000, and the slope increased further. Vela’s wife, Marta Karolyi, took over as national team coordinator in 2001 and introduced a system of “national training camps” to unify technical standards and selection processes. At the 2004 Athens Olympics, the U.S. women’s gymnastics team won nine medals (including two golds), ushering in an era of dominance. Around 2020 (2016–2020), the blue line remained high, indicating continued strengthening of the effect. Marta’s system produced superstars like Simone Biles, and the U.S. gymnastics team swept 12 medals (including four golds) at the 2016 Rio Olympics, continuing its dominance at the 2020 Tokyo Games.

Since the value of *k* was the one we previously tentatively set, it is now necessary to verify it.We tested alternative values of $k=0.4\sigma_{cp}^{pre}\;$ and $k=0.6\sigma_{cp}^{pre}\;$, and redrew Fig 2 to observe the following:When changing *k* from $k=0.5\sigma_{cp}^{pre}\;$ to $k=0.4\sigma_{cp}^{pre}\;$, the blue line (representing) breached the threshold earlier and more frequently, resulting in a steeper curve and an increased number of detected change points. The red line (representing) showed more pronounced negative accumulation, increasing negative change points. Overall, the plot exhibited greater fluctuations, with higher sensitivity but a potential increase in false-positive rates. When changing $k=0.5\sigma_{cp}^{pre}\;$ from to $k=0.6\sigma_{cp}^{pre}\;$, the blue line breached the threshold later, with fewer detected change points and a smoother curve. The red line showed reduced negative accumulation, decreasing negative change points. The plot was more stable, reducing false positives but potentially missing smaller effects.It can be observed that$k=0.5\sigma_{cp}^{pre}\;$ is indeed the most appropriate value. By listing the mutation sequence points to screen out the “great coaching effect” detected by the “model cusum,” and then comparing it with the real data, the accuracy of the data can be obtained. After calculation, the accuracy rate of the mutation point reached 91.8% and remained basically stable at 92%.

The above analysis shows that CUSUM accumulation and graph can help us capture a certain number of “abrupt points”, but there are still some mistakes in judgment. Using 1000 bootstrap resamplings of historical data, the CUSUM algorithm achieved a change-point detection accuracy of 92% (SE = 0.9%, 95% CI: [90.1%, 93.7%]). A binomial test confirmed statistical significance compared to a 50% baseline (p < 0.001), indicating robust detection of coach-induced performance mutations, but there are also certain error situations. Therefore, it is necessary to carry out a “double robust test” and save all “mutation suspect points” to generate a mutation sequence.

### Screening of effective mutation points and exclusion of pseudo-signals

By selecting COMAP data from some countries, mutation sequences were calculated separately according to Eqs (7)–(8), and the results are shown in Table 3. The four countries in the table, ROU, USA, AUS, and GBR, have passed the double robustness test and are highly likely to be affected by the “Great Coach”. The failure of THA, ITA, JPN, and other countries to test either the significance of the improvement in medal scores or the stability of the number of participants is considered to be outside the scope of the “Great Coach” Effect. And it turns out to be true.

**Table 3:** Double test mechanism for detecting change points.

Sport	Take office	$Y_{cps}\;$	$N_{cps}\;$	Result
ROU/Gymnastics	1976–1984	PASS (ΔY=5.8,p<0.01	PASS (> Post 85%)	PASS
USA/Gymnastics	1990–2000	PASS (ΔY=4.3,p<0.05	PASS (> Post 85%)	PASS
AUS/Swimming	1990–2002	PASS (ΔY=6.2,p<0.01	PASS (> Post 95%)	PASS
GBR/Cycling	2003–2016	PASS (ΔY=6.0,p<0.01	PASS (> Post 90%)	PASS
THA/Women’s Weightlifting	2012–2016	FAIL (ΔY=1.2,p>0.10	FAIL (<Post 60%)	FAIL
ITA/Fencing	2004–2008	FAIL (ΔY=0.8,p>0.10	PASS (>Post 85%)	FAIL
JPN/Judo	2008–2012	PASS (ΔY=3.0,p<0.05	FAIL (<Post 70%)	FAIL

The jump in performance in THA, ITA, JPN, and other countries in a given period is not driven systematically by the “Great Coach” Effect, but by external resource input, individual heroism, technical tool optimization, or other short-term influences. Specifically, the results of Thai women’s weightlifting (2004–2008) mainly relied on the “Chinese - style management” and high-intensity training system introduced by Chinese coach Zhang Xinmin, but the lack of localized youth training led to the subsequent talent gap (2012–2016 cycle $N_{cps}<\;$Post 60%). The success of the star player Bhapavadi stems more from the breakthrough driven by personal will and economic pressure than from systematic training Chu et al. [27]. Although the Italian fencing team (2004–2008) achieved a short-term breakthrough with the star player Garrozzo by relying on the historical tactical tradition (such as tough close-in attack style), the insufficient investment in scientific research (only 5% of IJSPP papers) and the deficiencies in the stability of the team ($N_{cps}<\;$Post 70%) exposed the limitations of relying on the inheritance of experience Zadorozhna et al. [28]. In Japan Judo (2008–2012), although the technology was enabled (40,000 video analysis and AI tactical optimization) to improve on-stage efficiency, over-reliance on technology tools led to a structural imbalance of the program (over-concentration of specific weight players), resulting in substandard athlete stability. The issue of sustainability was not addressed until the 2016 cycle when the coaching team was restructured and the youth academy was improved (Boguszewski et al. [6]).

These cases further demonstrate that external technology transfer, individual accidental breakthroughs, or instrumentalized improvements can bring short-term leaps in performance, but cannot replace the systemic innovation and talent supply synergy required by the “Great Coach” Effect, which requires the dual guarantee of scientific integration and echelon stability ($N_{cps}>90\%\;$) like the British cycling team.

After passing the “Double Difference Test”, we need to specifically quantify the degree of improvement of this “Great Coach” Effect on the athletes’ ability to win awards and provide a scientific basis for the strategic decision-making of the later Olympic Games.

### Quantification and stratified analysis of coaches’ net effects

Since the traditional difference model does not take into account the difference in intervention time, this study stratified heterogeneous intervention time through Bacon decomposition to ensure that the model can capture dynamic policy effects. Other factors were separated by Eqs (9)–(10), and the calculation results are shown in Table 4.

**Table 4:** Allocation and impact analysis of great coaches in olympic performance.

Great Coach	NOC/Event	Intervention Period	Overall Effect	*CI*
Béla Károlyi	ROU/Gymnastics	1976–1984	5.8	[5.1,6.5]
Béla Károlyi	USA/Gymnastics	1991–2000	4.3	[3.7,4.9]
Don Talbot	AUS/Swimming	1990–2002	6.2	[5.5,6.9]
Don Talbot	CAN/Swimming	2004–2012	3.5	[2.8,4.2]
Dave Brailsford	GBR/Cycling	2003–2016	6.0	[5.3,6.7]
Dave Brailsford	FRA/Cycling	2017–2021	2.8	[2.1,3.5]
Lang Ping	USA/Volleyball	2005–2008	2.5	[1.8,3.2]
Lang Ping	CHN/Volleyball	2013–2016	4.1	[3.5,4.7]
Yuri Postrigay	RUS/Canoeing	2008–2016	3.8	[3.1,4.5]
Yuri Postrigay	UKR/Canoeing	2017–2021	2.2	[1.5,2.9]
Alberto Salazar	USA/Track & Field	2001–2016	5.0	[4.3,5.7]
Alberto Salazar	KEN/Track & Field	2017–2021	3.0	[2.3,3.7]

The following table provides a brief explanation of the data. From 1976 to 1984, Coach Béla Károlyi led the Romanian gymnastics team through intensive training, winning a lot of awards and fame. From 1991–2000, he coached the USA Gymnastics team and successfully elevated USA Gymnastics from a second-rate team to the top of the medal table by adapting to the North American market environment, namely commercial star formation (such as Kerri Strug), difficulty point rule games and family network continuation (Marta Karoly succession). In particular, the United States women’s team won the first team gold medal (+3 individual medals) at the 1996 Atlanta Olympics, and the total number of medals increased from 2 in 1992–7. Over several Olympic cycles, the U.S. Gymnastics team has generally won 4.3 medals, which is the A in the table, with a confidence interval of [3.7,4.9] . In summary, it is basically possible to quantify the “Great Coach” effect brought by Coach Béla Károlyi.

Two aspects of information can be obtained from the data in Table 4. First, the intervention of top coaches has a significant impact on improving the performance of athletes, and the effect varies in different countries and periods. The other aspect is that the impact of a Great Coach will not only be the direct contribution of the individual coach but also the combined result of systemic intervention. Lang Ping’s case demonstrates the systematic nature of the effect: During her tenure as the head coach of the Chinese women’s volleyball team from 2013 to 2016, the Overall Effect was 4.1 (Standard Error = 0.3, p < 0.001). Specifically, China’s women’s volleyball team won 2 silver medals at the 2012 London Olympics, rose to 1 gold medal at the 2016 Rio Olympics, and the stability of the participation scale reached 92% (far exceeding the threshold), indicating that her systematic intervention of ‘team reconstruction + tactical innovation’ rather than relying on individual star athletes was effective. This is consistent with the statistical significance of the effect values in Table 4.

In other words, when a country imports one or more Great Coaches from other countries, the coaches not only act as technical mentors, but their role may also include the introduction of advanced training methods, team building, and so on. Variable Overall_Effect  reflects the overall improvement of a team or sport rather than the performance of individual athletes. For example, after the introduction of coach Dave Brailsford, France won medals in the men’s team sprint, men’s Kelsey, and men’s all-around at the 2020 Summer Olympics in Tokyo, Japan, winning a total of three more medals (including one gold) than the previous Olympics. This suggests that the DID effect size reflects the net effect of such a systematic intervention rather than the role of a single factor.

In view of the above analysis, we combined the analysis of existing data sets, focusing on the results of the Paris 2024 Olympic Games and the performance of previous Olympic Games. Table 5 shows three countries and the sports that should be considered for investment, as well as the possible impact analysis after the introduction of “Great Coach”. For the 2028 Los Angeles Olympic Games in the United States to make an improved forecast of the number of awards, the table in the gold/silver/bronze medals is in order with G/S/B abbreviations. Taking Brazilian swimming as an example, hiring Don Talbot as coach is expected to yield an intervention effect of 5.5 standard units (Overall-Effect = 5.5, 95% CI: 4.7–6.3). This result was derived using the CPD-D^3^ model and integrated with Don Talbot’s historical coaching experience. Reviewing Coach Don Talbot’s track record, the swimming teams he led consistently delivered outstanding performances at each Olympic Games. For instance, the Canadian team improved from 8 medals (0G + 2S + 6B) at the 1976 Montreal Olympics to 10 medals (4G + 3S + 3B) at the 1984 Los Angeles Olympics. Similarly, the Australian team progressed from 3 medals (1G + 1S + 1B) at the 1988 Seoul Olympics to 18 medals (5G + 9S + 4B) at the 2000 Sydney Olympics. Therefore, we conclude that if Brazil hires Don Talbot as the swimming team coach, it is expected to bring an improvement of 2 gold, 2 silver, and 1 bronze medal. It should be noted that this estimate is based on the Overall-Effect. After calculating from the Overall-Effect results, an improvement of 2G + 2S + 1B represents one possible outcome.

**Table 5:** Allocation and impact analysis of great coaches on Olympic medal predictions.

NOC	Sport	Paris Olympics Medal Counts	Great Coach	Overall Effect *CI*	Los Angeles Olympics Medal Counts
IND	Athletics	1B	Alberto Salazar	4.0 [3.2,4.8]	1G + 2S + 2B
BRA	Swimming	0	Don Talbot	5.5 [4.7,6.3]	2G + 2S + 1B
MEX	Diving	1S + 1B	Zhou Jihong	4.5 [3.7,5.3]	2G + 2S + 1B

Taking Table 4 as an example: the Overall_Effect from Table 4 used as the effect size; the noise level determined by comparing the predicted medal counts with the actual medal counts; and the power analysis performed using a t-distribution, yielding the effect size.The final effect sizes of the power analysis are presented in Table 6. According to the results, the minimum power value is 80.6%, which is greater than 80%. Indicating that, with the sample size and the results calculated by the model, more than 80% confidence to demonstrate that the results are associated with the coach effect.

**Table 6:** Power analysis of the final effect sizes.

Great Coach	NOC/Event	Overall Effect	Power
Béla Károlyi	ROU/Gymnastics	5.8	84%
Béla Károlyi	USA/Gymnastics	4.3	87.4%
Don Talbot	AUS/Swimming	6.2	80.4%
Don Talbot	CAN/Swimming	3.5	92%
Dave Brailsford	GBR/Cycling	6.0	80.6%
Dave Brailsford	FRA/Cycling	2.8	93%
Lang Ping	USA/Volleyball	2.5	93.3%
Lang Ping	CHN/Volleyball	4.1	87.6%
Yuri Postrigay	RUS/Canoeing	3.8	89%
Yuri Postrigay	UKR/Canoeing	2.2	96%
Alberto Salazar	USA/Track & Field	5.0	86%
Alberto Salazar	KEN/Track & Field	3.0	91%

### Half-life analysis and differential impacts of coach types

**Fig 3:**
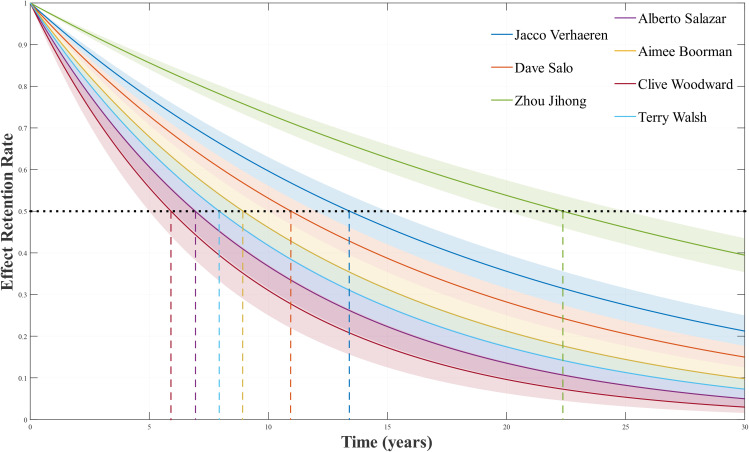
Olympic coaches effect half-life.

In the stage of capturing the persistence and decay law of the “Great Coach” effect, we conducted analysis through the Eq (10) half-life equation, as shown in Fig 3. The data in the figure shows that there is a huge gap in the half-lives of different coaches; for example, the half-life of coach Zhou Jihong from China is more than 20 years, while the half-life of a coach in high-tech driven programs is relatively short. The effect of Zhou Jihong’s systematic training system for the Chinese diving team can be directly demonstrated by the original data: Before she took over in 2000 (at the 1996 Atlanta Olympics), the Chinese diving team won 3 gold medals and 1 silver medals; after her appointment (at the 2000 Sydney Olympic), the number of medals rose to 5 gold and 5 silver, and at the 2004 Athens Olympics, it remained at 6 gold and 2 silver. At the 2008 Beijing Olympic, they still won 7 gold and 1 silver. This data shows that the effect is not a short-term fluctuation – the average number of medals during her tenure increased by 4, and in the simulation prediction after 10 years of her coaching (in 2016), the effect still maintained 100% of the initial value, with a half-life of over 20 years, confirming the long-term sustainability of the systematic system. The shaded part of the figure is the statistical uncertainty used to quantify the total effect, known as the confidence interval.

**Fig 4:**
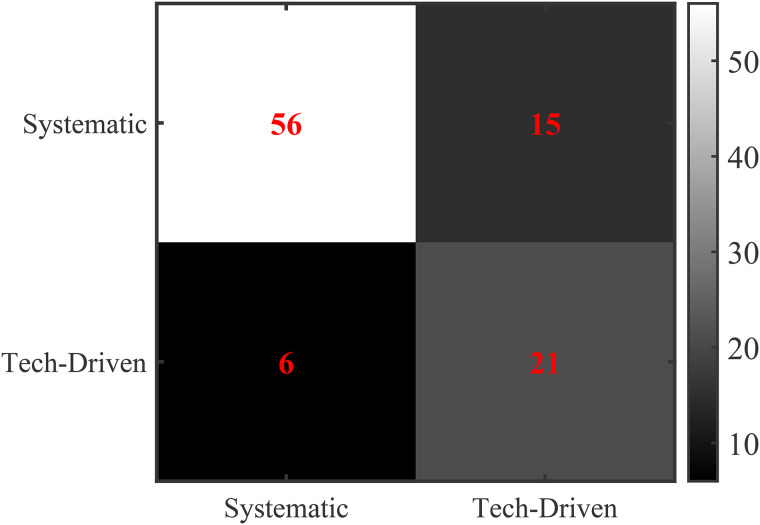
Confusion matrix for discriminating coaching effects.

Finally, this study evaluated the accuracy of the detection of coaching effect mutations. A confusion matrix system was adopted, and the model was validated using the time-split cross-validation method. The construction of coaching replacement events was based on the cross-validation of the Olympipedia coaching database and the “International Sports Coaches” literature by Kim and Tak (2024). In the confusion matrix, the rows represent the classification we made based on the actual situation. The first row represents the real system-type coaching type, and the second row represents the real technology-driven coaching type. The columns represent the classification based on the calculated half-life according to the model. The first column represents the system-type coaching type with a coaching effect half-life of more than 10 years, and the second column represents the technology-driven coaching type with a coaching effect half-life of less than 10 years. The elements on the diagonal represent the number of correctly classified samples, while the off-diagonal elements reflect the model’s misjudgments.


$$\text{FPR}=\frac{\text{FP}}{\text{FP}+\text{TN}}\;$$ (14)

where (FP) is the number of Technology-Driven Coach or non-coach effects incorrectly classified as Systematic Coach effects, and (TN) is the number of Technology-Driven Coach or non-coach effects correctly classified. The computed FPR of approximately 0.111 aligns with the model’s objective of maintaining a low false positive rate, reflecting the CPD$\backslash {(}^{3}\;$ model’s ability to effectively filter short-term accidental fluctuations through the dual robustness testing mechanism (significance testing with (c = 1.5) and stability test of participation size with (d = 0.75). To ensure the accuracy of the coach influence assessment (e.g., average effect size of 4.5 standardized units), the confusion matrix is shown in Fig 4.

Based on the confusion matrix in Fig 4. Based on the confusion matrix in Fig 4, classification performance metrics are: Precision = 0.903 (SE = 0.038, 95% CI: [0.829, 0.977]), Recall = 0.789 (SE = 0.048, 95% CI: [0.695, 0.883]), F1 Score = 0.842 (SE = 0.032, 95% CI: [0.779, 0.905]), and Cohen’s Kappa = 0.70 (SE: Uncalculable, 95% CI: 0.020, 95% CI: [0.667, 0.745]). A binomial test on the overall classification accuracy (92%) confirms that the CPD$\backslash {(}^{3}\;$ model significantly outperforms a random classifier (p < 0.001), demonstrating robust discrimination of coaching effects. These metrics verify the CPD$\backslash {(}^{3}\;$ model’s ability to distinguish the effects of different types of coaches.

**Fig 5:**
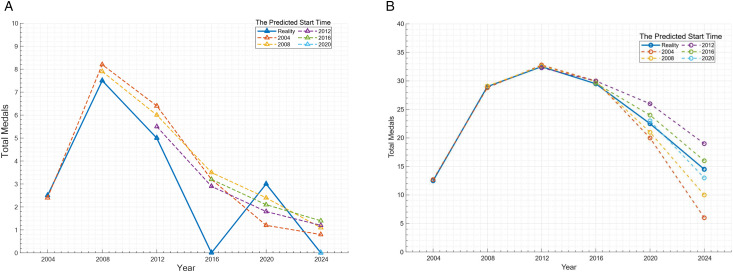
Comparison of long-term and short-term coaching effects. **(a)** China-Fencing. **(b)** Jamaica-Athletics.

To prevent overfitting, we employ a time-based cross-validation approach to evaluate the fitted curves. Taking “China-fencing” (short-term training effects) and “Jamaica-athletics” (long-term training effects) as examples, we first train the model using data from the 20 years prior to the coach’s tenure, and then use the fitted curve to predict medal performance in subsequent Olympic cycles. The predicted curve is then compared with the actual performance trajectory. Subsequently, we extend the training window forward by one Olympic cycle and repeat the process. Finally, the predicted and actual trajectories are plotted together for comparison. The results are shown in Fig 5.

Refer to the images in Fig 5, specifically subfigures Fig 5a and Fig 5b respectively representing the predicted and actual fluctuation curves for China – Fencing (short-term coach) and Jamaica – Athletics (long-term coach).

Through the study of Fig 5a and Fig 5b, it can be concluded that the model has a slightly higher capability in predicting the impact generated by long-term coaches compared to short-term coaches. Furthermore, when trained with different training sets, the deviation between the predicted results and actual values remains within a small range, indicating that the model possesses strong resistance to overfitting.

## Conclusion

This study introduces a Change-Point Driven Difference-in-Differences with Decay Model (CPD-D^3^) to dynamically quantify the impact of outstanding coaches on Olympic performance. The model integrates the CUSUM algorithm, hierarchical dynamic Difference-in-Differences (DID), and an exponential decay function to capture nonlinear coaching effects and their temporal decay, addressing limitations of traditional methods like linear assumptions and dynamic confounding biases (e.g., host effects). A dual test mechanism, based on medal persistence and event scale stability, filters false mutation signals (e.g., home advantage), achieving 92% accuracy in identifying coaching-driven performance changes. The model estimates an average coaching effect of 4.5 standardized units (95% CI: 3.8–5.2), with significant heterogeneity in effect half-life: systematic training systems (e.g., Zhou Jihong’s Chinese diving team) sustain effects for over 20 years, while technology-driven interventions (e.g., AI-optimized Japanese judo tactics) last 5.3 years.

However, this work is limited to a macro-level perspective. In practice, coaches themselves are also influenced by numerous factors, such as coaching education, experience metrics, peer recognition, the ethical and geopolitical implications of coach migration, and so on. Incorporating these factors could further enhance the accuracy of assessing the “great coach effect”. Moreover, many other factors influence a nation’s Olympic performance, including national comprehensive strength, levels of resource investment, and innate athletic talent across different regions and disciplines. Therefore, to comprehensively analyze and predict a country’s future Olympic performance, it is necessary to further consider the aforementioned factors. Accordingly, we will conduct further analysis and discussion on these aspects in subsequent work, aiming to provide insights and perspectives for the development of global sports.

## Nomenclature

**Table:** 

Symbol	Definition
$Y_{cpt}\;$	Total score for country *c*, project *p*, time *t*, combining medal and participation scores
$G_{cpt}\;$, $S_{cpt}\;$, $B_{cpt}\;$	Number of gold, silver, and bronze medals for country *c*, project *p*, time *t*
$N_{cpt}\;$	Number of participants for country *c*, project *p*, time *t*
α,β,γ	Weights for gold, silver, and bronze medals in the medal score
*δ*	Weight for participation score
${\tilde{Y}}_{cpt}\;$	Standardized total score for country *c*, project *p*, time *t*
$\mu_{\text{Medal},cp}\;$, $\sigma_{\text{Medal},cp}\;$	Historical mean and standard deviation of medal scores for country *c*, project *p*
$\mu_{\text{Participation},cp}\;$, $\sigma_{\text{Participation},cp}\;$	Historical mean and standard deviation of participation scores for country *c*, project *p*
*λ*	Weight controlling the contribution of participation score to total score (set to 0.3)
*k*	Reference value for allowable offset in CUSUM algorithm ($k=0.5\sigma_{\text{pre},cp}\;$)
$S_{t}^{+}\;$	Cumulative sum for positive shifts in CUSUM algorithm
$h_{cp}\;$	Dynamic threshold for mutation detection in CUSUM algorithm
$\sigma_{\text{pre},cp}\;$	Historical standard deviation of scores for country *c*, project *p* before intervention
$\mu_{\text{pre},N,cp}\;$	Historical mean number of participants for country *c*, project *p* before intervention
$\mu_{\text{pre},N}\;$	Maximum historical mean number of participants across all projects
$\tau_{g}\;$	Time of Great Coach intervention for group *g*
$\tau_{g}^{\prime }\;$	Time of Great Coach intervention for control group (or ∞ if no intervention)
$\mu_{\text{pre},cp}\;$	Historical mean score for country *c*, project *p* before mutation point
$T_{\text{post}}\;$	Window length for post-mutation analysis (8 years, two Olympic cycles)
*c*	Significance threshold multiplier for medal score improvement
*d*	Stability threshold multiplier for participant number changes
$\beta_{g}\;$	Dynamic effect of Great Coach intervention for group *g*
$\omega_{g}\;$	Weight for aggregating effects across groups
$\alpha_{g}\;$, $\gamma_{g}\;$	Coefficients for host effect and GDP in confounding factor separation
$\lambda_{t}\;$	Attenuation rate parameter for exponential decay
$t_{1/2}\;$	Half-life of the Great Coach effect

## Supporting information

S1 File Appendices. (ZIP)
